# Behaviour Therapy and Behaviour Modification Background and Development

**DOI:** 10.5334/pb.450

**Published:** 2018-07-26

**Authors:** Paul Eelen

**Keywords:** Behaviour therapy, learning theories

## Abstract

This manuscript is part of a special issue to commemorate professor Paul Eelen, who passed away on August 21, 2016. Paul was a clinically oriented scientist, for whom learning principles (Pavlovian or operant) were more than salivary responses and lever presses. His expertise in learning psychology and his enthusiasm to translate this knowledge to clinical practice inspired many inside and outside academia. Several of his original writings were in the Dutch language. Instead of editing a special issue with contributions of colleagues and friends, we decided to translate a selection of his manuscripts to English to allow wide access to his original insights and opinions. Even though the manuscripts were written more than two decades ago, their content is surprisingly contemporary. This manuscript is a transcription of a lecture that was published in 1980. It was Paul Eelen’s first public presentation after a two-year study in the United States, which has inspired much of his later thinking. The text can be viewed as a manifesto for behaviour therapy as it was then advancing in Belgium and the Netherlands.

This presentation was given as the introduction to the L.A.P.P. seminar day held October 27, 1980, which was themed: *Behaviour modification*. The lecture was published as: Eelen, P. (1980). Gedragstherapie en gedragsmodificatie: Achtergronden. *Leuvens Bulletin L. A. P. P., 39*, 1–19.

Krasner, one of the promoters of behaviour modification, published an eulogy in 1976 for its alleged death (Krasner, 1976). If it was a true eulogy, this seminar, is a posthumous tribute. But this eulogy was – luckily – pronounced at a time when the body was not identified. So, it was not known who or what had been buried. This brings us to one of the fundamental features of behaviour therapy: It is a house with many rooms, and, probably, there was never a prevailing approach from the start. Of course, names such as Wolpe, Eysenck, Skinner, Bandura, etc. are intrinsically associated with behaviour therapy. But none of them can be considered ‘Godfather’. Moreover, all of them represented a diverging view: Anyone who has followed the pointed altercation between Wolpe and Bandura (Wolpe, 1978), is probably surprised to hear that both are members of the American Association for Behavioural Therapy, and that both have had an enormous influence on the theory and practice of behaviour therapy and behaviour modification.

Before continuing the introduction to this seminar, it is useful to reflect on the distinction between behaviour therapy and behaviour modification. Both terms are often interchangeably used. Yet some distinguish between both terms, albeit not always for the same reasons. Behaviour therapy is sometimes considered a part of behaviour modification. The distinction is then based on a difference in population. Behaviour therapy involves the treatment of patients, as is the case in psychotherapy, whereas behaviour modification can also be used outside therapy. For other authors, the distinction can be traced back to a difference in theoretical foundation. Behaviour therapy is then relatively more informed by the Pavlovian learning paradigm, whereas behaviour modification is more associated with the operant learning paradigm, in particular the Skinnerian school of learning. This distinction probably makes sense because Skinner and his followers do have a unique methodology. Nevertheless, in this presentation both terms will be used synonymously as most handbooks do. Moreover, let us not forget that Skinner was one of the first to use the term ‘behaviour therapy’.

Evidently, an introduction to a theme necessitates selection. After a general contextualisation of behaviour therapy, I will limit myself to a few comments on the primary characteristics of this approach. It will not be possible to explore the various applications that have emerged from this approach, which vary from addressing pollution problems to the treatment of patients with psychosis. The practice of behaviour modification will only be briefly discussed. The afternoon sessions will offer ample time and space to explore these aspects.

## Attempt to develop an acceptable definition or description

When behaviour therapy originated – some 25 years ago – its identity was formed by opposing the conventional psychotherapeutic practices, which were dominated by the psychoanalytic model. The breakthrough of behaviour therapy was undoubtedly fostered by the discomfort with the psycho-analytical paradigm: both in terms of its theoretic underpinnings and its clinical efficiency.

As regards clinical efficiency, Eysenck’s famous article in 1952 ‘*The effects of psychotherapy*’ was a clear blow below the belt. With data that were – at first glance – indisputable, he demonstrated that traditional psychotherapy – whatever it means – had null effects. To be more precise, he provided evidence that neurotic patients fare just as well with as without therapy. What is striking when rereading this article today, is its rather moderate tone (especially for someone like Eysenck). Still, in the eyes of Bergin (1971), this article marked the beginning of ‘two decades of vitriol’. Not surprisingly, it prompted a series of emotional reactions. Nobody likes to see evidence that what they do, has no effect. In 1966 – 14 years after his first article – Eysenck still backed the same conclusion. In the meantime, he had further fuelled the polemics by demonstrating that behaviour therapy did have effects. When Bergin wrote a paper on the ‘The evaluation of therapeutic outcomes’ in 1971, he had probably hoped that his paper would only be of historical importance, and would provide a glance from a distance on the polemics that Eysenck had created. At the same time, he demonstrated the complexity of outcome research –also using data. A similar conclusion was reached in behaviour therapy. The well elaborated book of Kazdin and Wilson (1978) is just one example. Nevertheless, Eysenck did not change his opinion. Only a year ago, he confirmed that nothing in the literature provided evidence to reject his null-hypothesis (Eysenck, 1978). The polemic will probably never end, at least not for Eysenck. What is clear today is that the original triumphalism – “the behaviour therapy is the only truly effective and efficient form of therapy” – is only endorsed by few. It is, however, undoubtedly the case that treatment goals are more articulated in behaviour therapy. When the ‘outcome’ question is formulated in terms of the achievement of treatment goals, extensive literature reveals that behaviour therapy is not doing a bad job. However, it remains to be debated what treatment goals exactly are.

As regards the theoretical underpinnings, it was almost standard practice in this early phase to situate and define behaviour therapy by contrasting it with other approaches, with psycho-analysis first and foremost. Usually Eysenck’s schematic overview (1959) was used to shed light on the differences between both approaches, in which he advanced a pronounced preference for behaviour therapy. However, it is seldom asked what the heuristic value of such comparisons is. These comparisons lack any persuasive power. For me personally, they bring to mind the introductory works to philosophical schools, where for example, Hegel’s system is criticized in a few paragraphs leading one to conclude that Hegel was a ‘minor habens’. Freud once claimed that his theory could only be evaluated by those who were initiated in it. This, of course, provided extra ammunition to his opponents. But his statement probably contains some truth. Some behaviour therapists have used the same argument.

The almost dogmatic aversion to anything that might be associated with the conceptual framework of psychoanalysis manifested itself in many ways. For example, Dollard and Miller’s (1950) ‘Personality and Psychotherapy’ work, which was a monumental attempt at integrating learning psychology and psychoanalysis, never became popular. It was not taken seriously by psychoanalysts because it was almost an insult to extrapolate findings from rats to humans. Just imagine a rat with an Oedipus complex! It was equally inacceptable to behaviour therapists because it appeared too much as a translation of psychoanalytic concepts into learning terms, and because it had little to bear on psychotherapeutic practice. It mainly offered a new vocabulary but did not inspire novel intervention methods. Even more so than with Dollard & Miller, the aversion was exemplified in the depreciation for Masserman’s work (1943) on experimental neurosis in cats. His experiments directly inspired Wolpe (Wolpe, 1958), whose experiments – by the way – were of a far lower standard in terms of methodology and data analyses. Unfortunately, Masserman had used a vocabulary that was strongly informed by psychoanalysis. His focus was on the notion of ‘conflict’, operationalised in terms of aversive stimulation contingently following approach behaviour for food. In his experiments, Wolpe wanted to demonstrate that so-called neurotic reaction patterns can develop independently from any conflict, but simply by confronting the animal with a series of aversive stimuli (i.e. electric shock). This proved to be the case. However, Wolpe merely demonstrated that conflict is not a necessary condition. Hence, this finding does not rule out the possibility that conflict is a sufficient condition for the establishment of so-called neurosis. It is striking that the notion of ‘conflict’ is again used in recent behaviour therapy literature. Finally, I give one last example to illustrate the anti-psychoanalytic reflex in this early period. In implosive therapy, introduced by Stampfl and Levis (1967), the patient is ‘bombarded’ with the core features of his fear. Stampfl was a psychiatrist with psychoanalytic training, and Levis had been a student of Spence. A ‘happy marriage’ at first sight (Levis, 1970). Lewis searched and found a fairly adequate experimental paradigm, but his view was hardly noticed in behaviour therapy because it relied too strongly on Freudian concepts.

All these anti-reactions probably may have been justified in the beginning: Others had to be persuaded of one’s merits. But, by now, the contribution of behaviour therapy is sufficiently established. Behaviour therapy is now expected to define itself in positive terms (‘What it is’) rather than in negative terms (‘What it is not’). Wolpe (1976) believed that the following definition was correct: Behaviour therapy is the whole body of “treatment methods derived from experimentally established principles and paradigms of learning (and related principles)”. Those who do not concur with this view, Wolpe called ‘malcontent’. Kirsch (1977) considered this definition as too restrictive and too dogmatic. According to him, behaviour therapy can only be defined as that what is done by therapists who identify themselves as behaviour therapists. The question is whether Wolpe’s description is actually too restrictive and dogmatic. In my view, his definition was critically commented upon because of what was believed that Wolpe deeply thought than because of what he actually wrote. As it is, his verbatim description closely followed the one of the “American Association for Behavior Therapy”: “Behavior therapy involves primarily the application of principles derived from research in experimental and social psychology for the alleviation of human suffering and the enhancement of human functioning. Behavior therapy emphasizes a systematic evaluation of the effectiveness of these applications …” (Franks & Wilson, 1975, p. 2). Such definition can hardly be called restrictive or dogmatic. In fact, the reverse is true!

In 1969, Bandura already pleaded for the abandonment of the term ‘behaviour therapy’ because it was an “ill-defined partisan label” (Bandura, 1969). Probably, some day brand names for interventions will become useless, but it is highly unlikely that this will happen in the near future: At present there is a proliferation of conceptual systems, each with their own ‘label’, and, sometimes, also with their own dogma’s. Hence, the need to delineate behavioural therapy as an approach which – in principle – aims to apply insights, findings and methods from experimental psychology. Evidently, one should keep in mind that such approach only offers partial insight into the complex phenomenon of one human being influencing another human being in some way (i.e. therapy).

## A couple of general characteristics

In his “History of Behaviour Modification”, Kazdin (1978) identified four characteristics, which can be found more or less in most handbooks. I want to discuss these characteristics. The first characteristic will receive the most attention because it contains the core of the development as well as the current practice of behaviour therapy.

### The assumption that abnormal or problem behaviour is learned

This statement, when formulated in such a general way, is not unique for behaviour therapy. The psychogenic nature of many disorders is and remains one of Freud’s fundamental contributions. What is characteristic for behaviour therapy then is that it puts normal behaviour at the same level as abnormal behaviour, and that it assumes that both are initiated or maintained via similar learning principles. When behaviour therapy started off, learning paradigms were used to define these learning principles. They offered an acceptable frame of reference to describe the antecedent and consequent factors that influence behaviour.

It has become common practice, almost a caricature of behaviour therapy, to underline that the wisdom and techniques of behaviour therapy stems from experiments with animals, in particular rats and pigeons. Noteworthy, it is not so much the extrapolation from humans to animals that elicits resistance and opposition. For example, ethological approaches to abnormal behaviour are accepted with much more generosity. Rather, the experimental nature and particular language of conditioning seem to be rejected. The simple word ‘conditioning’ is an aversive stimulus for many, in the same way as the term ‘cognition’ is for some behaviour therapists. To a large extent, confusion has been raised by not sufficiently making a distinction between the procedures for conditioning experiments, the effects of these procedures, and the theoretic assumptions proposed to explain these effects. For example, calling a phobic reaction a conditional or conditioned response assumes, at most, an analogy between both phenomena. Fundamentally, any explanation of both phenomena remains possible, but nevertheless this analogy has heuristic value. When it is established – as a fundamental law – that a conditioned response disappears as a result of presenting the conditional stimulus only, it suggests an action plan. In addition, it suggests a notion of extinction, which possibly explains this law. However, also extinction is the result of a procedure and not an explanatory mechanism. To put it frankly: The question of why Pavlov’s dog salivates upon hearing a bell announcing the food remains open. Evidently, several explanatory models have been put forward, but most have become obsolete even though they occasionally re-emerge in the literature. For Wolpe, for instance, learning psychology ended with Hull, but not everyone in behaviour therapy agrees with this position.

Nonetheless, learning paradigms continue to play an important role in the theory and practice of behaviour therapy. Therefore, it remains valuable to reflect on their importance. We will focus on two issues: (1) Which question underlies the procedures of these paradigms? (2) To what extent are they relevant for clinical practice?

1) The value and limitations of conditioning models is determined by how we appreciate the fundamental questions underlying the used procedures. Take the example of *classical conditioning*: Essentially, it concerns the question of the conditions under which an organism learns an association between two events. It is a historic coincidence that Pavlov used a physiological index for this type of learning. Any other index would equally do, as long as it is demonstrated that the behaviour change can be attributed to the induced relation between the two stimuli. The historic coincidence, however, has had far-reaching consequences for the way classical conditioning has been represented. The physiological response has become isolated from the full event, the event of an organism learning a new relationship. Fortunately, this is changing. I will briefly illustrate this. Jenkins, a leading experimentalist in the field, published last year a study with the simplest design imaginable: A large room in which a lamp announces the delivery of food to a dog who freely moves around that room (Jenkins, et al. 1978). Careful observation of the dogs’ global behaviour was used as the dependent variable. Seventy years after Pavlov’s first findings, this experiment might at first sight prove that this field repeats itself in an almost ridiculous way. Upon scrutiny, however, this experiment shows that we still know very little about what is actually happening. Identifying the necessary and sufficient conditions to learn associations is the first step towards a theory. For a long time, the temporal contiguity of two stimuli was viewed as the only necessary – and frequently also sufficient – condition. Therefore, the idea was that one could rely on rather simple mechanisms to develop a theory. The overall picture that emerges from such explanatory models is that of a passive organism in which associations and connections automatically strengthen. However, by now it is obvious that temporal contiguity is neither necessary nor sufficient. One of the most recent theories on classical conditioning has been proposed by Rescorla and Wagner (1972). The intuition that lies at the heart of this theory can be translated into psychological terms as follows: As soon as something happens to the animal – and this ‘something’ is a biologically relevant event such as food or pain in most experiments – which has not yet been predicted by another stimulus or a context, it is as if the animal searches for a predictor of this unexpected event. This is a fundamental why-question. It could thus be said that classical conditioning is fundamentally concerned with the question of how an animal construes a predictable world or – to put this differently using the words of Tolman & Brunswik (1935) – the focus is on the development of the ‘causal texture of the environment’. Operant learning can, mutatis mutandis, be considered as a procedure through which one studies how an organism learns a relationship between its behaviour and a particular outcome. The question here is how the organism construes a world and environment that can be perceived as controllable. The notions of ‘predictability’ and ‘controllability’ create a different frame of reference to describe both learning paradigms. They become increasingly more important, not because they sound nicer but because they create more room for a description and explanation of what is happening.

2) This brings us to the second question: what is the relevance of these learning principles as a model for behaviour modification? They have been used – and sometimes misused – to (a) explain the development of abnormal behaviour, (b) disentangle the factors that influence behaviour, and (c) develop particular therapeutic interventions.

(a) Although assessing the *aetiology* of the behaviour was less of a concern in the beginning of behaviour therapy, there was an emphasis on the usefulness of conditioning principles to explain the development of behaviour disorders. For classical conditioning, Watson & Raynor’s famous experiment (1920) with little Albert served – and still serves – as the primary model. This experiment is probably well-known, so only its essential elements are described here. Each time little Albert – an 11-month old baby- touched a white baby rat, a strongly aversive sound followed. In this way, Watson and Raynor wanted to demonstrate that phobic reactions can be rooted in conditioning experiences. After a while, it was noticed that the baby started to respond fearfully as soon as he saw the baby rat, and that this fear generalised to all sorts of similar objects. The scope of this experiment, however, has been greatly exaggerated. It certainly cannot be considered a prototypical explanation of the development of phobic behaviour. Also other aspects of this so-called experiment do not justify its assumed importance (Harris, 1979). In addition, it is routinely overlooked that replicating the experiment did not succeed (apart from the question to what extent one should even try to replicate such an experiment). A similar objection can be made regarding the use operant learning models to explain the development of abnormal or problem behaviour. We now refer to the famous study by Ayllon et al. (1965). A chronic, psychotic patient was made to hold a broom, and to carry this broom everywhere with her, and this for a long period. This behaviour was learned by delivering cigarettes – the reinforcer – contingent upon the execution of this bizarre behaviour. Two psychiatrists who were unaware of this learning history were subsequently invited to provide a diagnosis and explanation while observing the patient through a one-way screen. Not surprisingly, their interpretation deeply contrasted with the actual reasons. One of the psychiatrists identified the broom as a phallic symbol. However, who is the fool here? The patient with the broom, the people who designed the experiment, or the psychiatrists who agreed to provide an interpretation? I would like to highlight the role of the psychiatrists by drawing the following analogy: Assume we have a video of someone peeling onions, his – or her – cheeks full of tears. If you could only see his or her face, and had no knowledge of the context, what would be your interpretation? One can only generate hypotheses based upon the frame of reference that one is familiar with. Evidently, it is a precarious exercise to make a diagnosis based upon fragmentary observations. But the researchers are also not innocent. Not so much because of ethical objections (a patient had to carry a broom for an entire year), but because of the following remark: “The etiology of so-called psychotic symptoms exhibited by hospitalised patients or those in need of hospitalisation does not have to be sought in the obscure dynamics of a psychiatric disturbance.” In other words, because a so-called symptom can be created through operant principles, any other explanation would be superfluous. This is an obvious logical fallacy: Symptom A was caused by factor X under specific conditions; So factor X always causes A (Davison, 1969). A similar logical fallacy is at stake when the successful application of a technique based on learning principles is used to conclude that behaviour was originally learned through those same principles. In fact, not many advances have been made to explain the aetiology of problem behaviours using learning principles. In fact, the idea has started to emerge that many problems may develop outside the realm of learning principles. Here, I want to come back to the notions of unpredictability and uncontrollability. It is a fascinating hypothesis that the essential feature of all procedures involved in creating a so-called experimental neurosis – we refer to the studies by Pavlov, Maier, Masserman, Wolpe, Seligman, et al. – is that the animal either loses predictability or controllability of its environment (Mineka & Kihlstrom, 1977). This might mean that a fertile ground for the development of disorders lies in those situations where no adequate cause is found for a particular event, or when one has lost grip on its environment. Also this is essentially a learning process, but in these cases learning is about the fact that there is no unequivocal relation between events, or that there is no relation between what one’s behaviour and what follows. In such situations, one might continue to search for a cause, or to attempt to obtain control. In that sense, a person with a phobia towards a particular situation, or a compulsive person involved in excessive controlling may both have found a solution for their intrinsic tension. Are we here far or not far removed from a psychoanalytic interpretation that a disorder essentially is about failing of ‘normal’ adjustment mechanisms?

(b) A second observation on the usefulness of learning paradigms and concepts concerns the identification and description of the factors that maintain problem behaviour. This is actually ‘functional analysis’, which is considered the back bone of any attempt at changing behaviour. Systematic observation and questioning are used to identify the antecedent or consequent factors that maintain the problem behaviour. Based on this functional analysis, in which one acts as an experimenter, a hypothesis is advanced from which an intervention technique is derived. In that respect, a functional analysis is not at all simple. There is no recipe book that prescribes treatments for disorders. The language from conditioning paradigms is often adopted in functional analysis, using words such as discriminative stimulus, conditioned emotional response, reinforce, etc. And ‘coverants’, a term introduced by Homme (1965) as a contraction of ‘covert operants’, is used to represent cognitions. This vocabulary may seem odd, and it may indeed be too simplistic when describing real life problems. Obviously, these terms are reductionist, but the key question is whether these schematic presentations have any heuristic value. The latter is not determined by the vocabulary, but by the adequacy of the functional relationships. This *functional approach* to behaviour probably differs thoroughly from a more ‘understanding’ approach. A child that does not want to go to school will by most be labelled with school phobia. Here, a behaviour therapist will take a different approach from someone who looks at this event through, for example, psychoanalytic glasses. The latter will probably offer interpretations along the lines of separation anxiety from the mother. A behaviour therapist is less likely to draw such inferences and will more likely focus his analysis and action plan on the specific problem. This might result in an analysis of the mother-child relation, but this is not necessarily so.

(c) A last observation concerns the application of learning principles in the elaboration of a technique or intervention method. In an influential article, London (1972) argued that it is illusory to present these techniques as if they go back to learning theory. Such a statement, however, identifies again a procedure with the explanation that has been offered for the effects of this procedure. After all, there simply is no unequivocal learning theory. It is obvious that in the design and application of these techniques, one has been too strict in relying on procedures that were used with animals. The use of identical procedures does not mean at all that identical psychological processes are targeted, and it is only through targeting specific processes that a procedure ultimately works. Let me illustrate this with an example. Electric shocks are frequently used in animal research. A neutral stimulus can be rendered aversive to the animal through association with an electric shock. A similar procedure has been used in some behaviour therapy techniques, e.g. in alcoholism treatments, in the hope that the same result would arise. I immediately wish to add that the use of such techniques is rare, although in the public opinion such techniques are considered quintessential for behaviour therapy. It is, however, overlooked that an electric shock is a completely different event for an animal than for a person. The traumatic quality of a shock to an animal probably not only lies in the pain, but also in the complete incomprehensibility of what is happening. This is why a discrete and typically novel stimulus that coincides with the shock in time and space is experienced as one event by the animal. This is completely different for an adult human who knows that an electric shock is not intrinsically linked to the sight of e.g. an alcoholic beverage. The person only knows that the shock is conditionally connected to a particular stimulus under certain circumstances, e.g. in the clinic. In other words, this brings us to the fundamental question of attribution. As a consequence, the key issue in using such procedures is not to explain why they are not working, but instead why they are working under specific circumstances.

### Behaviour therapy is an application of findings from experimental psychology

A second characteristic mentioned by Kazdin is that behaviour therapy and behaviour modification aim to be an application of findings from experimental psychology. This is more wishful thinking than actual reality. How could it be otherwise? Who dares to claim to have an overview of what experimental psychology has accomplished and which parts are relevant? It is barely possible to keep up with the literature in one’s own domain. So, behaviour therapy will never be what it claims to be, but in doing so it remains in principle open to any new approach. As our discussion of the preceding characteristic has shown, behaviour therapy has all too often based its wisdom on a limited part of the conditioning literature, and has sometimes failed to take into account new theoretical evolutions. Authors like Bandura have greatly contributed to broadening the framework into what is called ‘social learning theory’. The conceptual framework has largely expanded, while not always providing clarifications to the same extent (Bandura, 1977a). In that context, it is remarkable that behaviour therapy, just like experimental psychology in general, is increasingly relying on a vocabulary from cognitive psychology. A huge shift has taken place in learning psychology: from the study of behaviour change to the analysis of memory and cognitive processes. Today the focus primarily lies on the study of the processes through which information is perceived, processed and recalled. Whether this cognitive psychology will make a contribution to behaviour change will largely depend on how well it succeeds in translating these intermediating processes into behavioural terms. The question of whether therapy or intervention should modify cognitions or behaviours, is probably a false one, resulting from creating a dichotomy between both terms. After all, it is only possible to talk about cognitions in terms of a process, of what the organism is doing. In his recent self-efficacy theory, Bandura (1977b) claims that every behaviour change is in fact dependent on a change in cognition. In his view, the central cognition can be summarised with one term, the efficacy expectancy, or the expectation one is able to effectively execute a particular behaviour. Yet, according to Bandura the most efficient procedures to impact this intermediary cognition are heavily relying on the actual execution of that behaviour. Empirical verification of such a claim ultimately comes down to correlating two types of behaving: at the verbal and at the performance level. This again brings us close to the theme of last year’s seminar: the relationship between attitude and behaviour.

### Direct focus on the behaviour

A third characteristic, according to Kazdin, is the direct focus on the behaviour one wants to change. This characteristic, too, has prompted a large number of comments. Behaviour therapy and behaviour modification would supposedly remain superficial, and not penetrate the real causal and intrapsychic determinants of the behaviour. In such comments, we can distinguish several components. First, what is frequently meant by ‘superficial’ is that behaviour therapy is ahistorical. In other words, the focus is solely on the ‘here and now’. This is both correct and incorrect. The aim is indeed to assess what the *actual* determinants of the behaviour are, but an extensive anamnesis often shows that the past continues to be actualised in one or another way. For emotional problems, it is often the case that the initial or main problem is only that what the patient is able to verbalize at a particular moment.

Undoubtedly, the comment also touches upon the fundamental issue of symptom substitution: Only changing external behaviour without reaching the deeper cause would result in the development of other symptoms. A lot has been written about this issue and it is impossible to summarize this debate in a few words. Let us repeat just this: symptom substitution is a hypothesis that first and foremost stems from a particular theoretical conceptualization of problem behaviour. To validate this hypothesis, one needs to clarify what a symptom is, and also when and in what form a substitution will take place. Failing to do so, novel problem behaviour can equally well be captured with other explanations. Bandura (1969) correctly observed that the problem of symptom substitution would never have been articulated in its current misleading form, if it was generally acknowledged that ‘behaviour’ is never eliminated in a vacuum (except through neurophysiological interventions), but always through (intentional or unintentional) manipulations of the factors that control this behaviour. It is of course possible to have different opinions on what those controlling factors are, but these differences in opinion can also occur amongst behaviour therapists. If a functional analysis hypothesises that a particular behaviour is a typical form of avoidance behaviour, the intervention will focus on the hypothetical cause of avoidance. In that case, it is not the behaviour itself that is targeted but rather the hypothetically assumed fear that sustains it.

### Methodology

A final characteristic is the experimental methodology of behaviour therapy and behaviour modification. This was already highlighted throughout our discussion of the previous characteristics. As Brinkman (1978) writes, behaviour therapy and behaviour modification includes an empirical cycle. Starting with an as precise as possible behavioural observation, a hypothesis relating to the factors that influence this behaviour is formulated, and based on this analysis an intervention selected. Eventually a systematic evaluation of the intervention follows. The application of operant learning principles has contributed to an extensive N = 1 research methodology. In addition, a wealth of experimental literature using analogous samples exists. Studies to assess the value of certain intervention techniques using subjects with e.g. a fear of snakes or spiders, or with a fear of public speaking, are well-known. It is remarkable, however, that several authors are opposed to this. Wolpe, for instance, rejects most of the criticisms on systematic desensitisation as irrelevant because they primarily relies on findings from studies with analogous populations (Kazdin & Wilcoxon, 1976). Some of this opposition probably stems from the fact that a majority of these studies demonstrate that several aspects of his technique are not necessary, and that the theoretical explanation offered for them is incorrect. Marks (1978), too, believes that research on clinical interventions is only valid when it is performed on clinical samples. It is impossible, however, to further this debate as long as it is not clearly specified what is typical about a so-called clinical population. If it is claimed that a critical and qualitative difference between so-called normal and abnormal behaviour exists, one of the fundamental assumptions of behaviour therapy is implicitly rejected. By the way, is the leap from so-called analogous to clinical populations larger than that from a neurotic cat to a neurotic human?

## Final observations

I am well aware that I have only sketched a brief picture of what is understood by behaviour therapy or behaviour modification. On some occasions, I may have played the devil’s advocate a bit too much. Somehow, I considered this my task in introducing this seminar.

Allow me, then, to conclude with a personal plea for the contributions of behaviour therapy. I do not have abundant clinical experience. But behaviour therapy, for me, will continue to bring to mind the patient I was allowed to treat during my training with Wolpe in Philadelphia. She was a 70-year-old woman who had suffered from agoraphobia for more than 30 years and who had been declared a hopeless case by several psychiatrists. After a couple of months of behaviour therapy intervention, she undertook several flights to New York at her own initiative, and she is currently on a cruise trip to the Caribbean islands. Let me put the psychoanalysts among you at ease: She sends me platonic love letters every now and then!

Such experiences of course do not offer proof of the value of behaviour therapy. Any person can probably come forward with remarkable successes. And when one gives this success deeper consideration, one inevitably asks oneself: what happened here? Anyone who is confronted with people in need in their daily practice, or anyone who wants to clarify certain data through empirical research knows all too well that we know far too little about what is the object of our study: human behaviour.

Allow me to close with a prayer in view of this ignorance. This is probably a unicum: concluding a presentation about behaviour modification with a prayer! I discovered it thanks to the dean of this Faculty, who hung it next to his door. I recommend this as a ‘cognition’ that should be present in all those who are engaged in behaviour modification:

God, give me the serenity to accept what I cannot change.
Give me the courage to change what I can change.
And most of all give me the wisdom to know the difference.
